# Genome-wide analysis of the common bean (*Phaseolus vulgaris*) laccase gene family and its functions in response to abiotic stress

**DOI:** 10.1186/s12870-024-05385-x

**Published:** 2024-07-19

**Authors:** Tong Cheng, Chunyuan Ren, Jinghan Xu, Huamei Wang, Bowen Wen, Qiang Zhao, Wenjie Zhang, Gaobo Yu, Yuxian Zhang

**Affiliations:** 1 College of Agriculture, Heilongjiang Bayi Agricultural University, Daqing, Heilongjiang China; 2 National Coarse Cereals Engineering Research Center, Daqing, Heilongjiang China; 3 College of Horticulture and Landscape Architecture, Heilongjiang Bayi Agricultural University, Daqing, Heilongjiang China; 4 College of Life Science and Biotechnology, Heilongjiang Bayi Agricultural University, Daqing, Heilongjiang China

**Keywords:** Laccase gene family, *Phaseolus vulgaris*, Abiotic stress, Development

## Abstract

**Background:**

Laccase (LAC) gene family plays a pivotal role in plant lignin biosynthesis and adaptation to various stresses. Limited research has been conducted on laccase genes in common beans.

**Results:**

29 LAC gene family members were identified within the common bean genome, distributed unevenly in 9 chromosomes. These members were divided into 6 distinct subclades by phylogenetic analysis. Further phylogenetic analyses and synteny analyses indicated that considerable gene duplication and loss presented throughout the evolution of the laccase gene family. Purified selection was shown to be the major evolutionary force through Ka / Ks. Transcriptional changes of PvLAC genes under low temperature and salt stress were observed, emphasizing the regulatory function of these genes in such conditions. Regulation by abscisic acid and gibberellins appears to be the case for *PvLAC3*, *PvLAC4*, *PvLAC7*, *PvLAC13*, *PvLAC14*, *PvLAC18*, *PvLAC23*, and *PvLAC26*, as indicated by hormone induction experiments. Additionally, the regulation of *PvLAC3*, *PvLAC4*, *PvLAC7*, and *PvLAC14* in response to nicosulfuron and low-temperature stress were identified by virus-induced gene silence, which demonstrated inhibition on growth and development in common beans.

**Conclusions:**

The research provides valuable genetic resources for improving the resistance of common beans to abiotic stresses and enhance the understanding of the functional roles of the LAC gene family.

**Supplementary Information:**

The online version contains supplementary material available at 10.1186/s12870-024-05385-x.

## Introduction

Laccase, a member of the multicopper oxidases (MCOs), functions as a p-diphenol–dioxygen oxidoreductase (EC.10.3.2) and is found widely among organisms, including bacteria, fungi, insects, and plants [1–4]. Laccase has been initially found to be an oxidase in Japanese lacquer trees and later identified as an oxidase containing metal ions [5, 6]. Laccase protein structure comprises three conserved binding regions: Cu-oxidase, Cu-oxidase_2, and Cu-oxidase_3 (PF00394, PF07731, PF07732) [7]. And the presence of a type 1 copper, a type 2 copper, and two type 3 copper atoms has been revealed as integral to the crystal structure of laccase, facilitating electron transfer essential to the catalytic process [6, 8, 9]. Although laccases mainly target aromatic compounds, which can also oxidize non-aromatic substrates, suggesting that laccases function in many ways due to the broad substrate [10–12]. The established catalytic mechanism involves type-1 Cu extracting electrons from the substrate, leading to substrate oxidation and radical production, which trigger numerous enzyme-independent secondary reactions [13]. Thus, laccases play a crucial role in a variety of biological processes. In fungal, laccases have been widely studied to be utilized in wastewater treatment, dye decolorization, and environmental lignin degradation [14–16]. In insects, laccase is crucial to exoskeleton formation [17]. In plants, known as copper-containing glycoproteins, laccases facilitate the oxidation of lignin monomers to radicals, playing a significant role in lignin biosynthesis and the construction of plant cell walls [18–20]. Despite the acknowledged importance, the analysis of practical roles of laccases in plants is full of challenge owing to the membership in a vast, redundant multigene family. In recent years, the rise of research has revealed some characteristics and biological function of laccase gene family [21]. The gene family has been identified in various plant species, including model organisms like *Arabidopsis thaliana* [22], *Oryza sativa* [18], *Gossypium spp* [23], *Glycine max* [5], *Camellia sinensis* [24], and *Brassica napus* [25].

The role of plant laccases in lignin biosynthesis and plant stress responses has obtained increased attention in recent years. Lignin is essential for enhancing plant tolerance and vitality. The overexpression of *LAC10* from rice in Arabidopsis has resulted in increased lignin accumulation in Arabidopsis roots, enhancing copper stress tolerance, due to reduced copper absorption enabled by increased lignification in the roots [18]. Conversely, the loss of function of the laccase genes *LAC17* and *LAC14* in *Arabidopsis thaliana* leads to reduced lignin content compared to the wild type, demonstrating the role in lignin polymerization and degradation [26]. Additionally, laccase participate in plant response to multiple stresses on account of the antioxidant and other physiological and biochemical functions. In *Camellia sinensis*, multiple laccase genes have been revealed to respond significantly to various biotic and abiotic stresses, including cold, drought, insects, and fungi, and playing roles in defense mechanisms against herbivores and potentially acting as detoxifying enzymes [24, 27]. In *Brassica napus*, laccase genes display distinct expression patterns in response to diverse stresses such as drought, extreme temperatures, and fungal infections [25].

Common bean (*Phaseolus vulgaris*) is an annual herb of Leguminosae, Papilionoideae, *Phaseolus* [28]. Common beans are globally consumed for the high level of dietary fiber and potassium that contribute to enhance satiety, as a crucial and nutritious food source for human health [29, 30]. Common beans are used as the staple food in many countries in South America and southeastern Africa [31]. However, recent societal developments and global climate changes have increased plant vulnerability to stresses such as low temperatures and herbicides [32, 33]. Low temperature (0℃-15℃) has been reported to limit the growth and development of common beans. In the seedling stage, sudden exposure to low temperature (4℃) could cause oxidative damage to the cell membrane and the photosynthetic system [34]. Herbicides often exceed the recommended dose in actual production, which leads to the pollution of herbicide residues in the soil, resulting in a decrease in the emergence rate of common beans, growth retardation, reduction in the accumulation of photosynthetic pigments and photosynthetic products, and the cultivation of common beans is seriously affected [35–38]. Laccase genes play a vital role in the plant response to abiotic stress, while the specific function and molecular mechanism of the laccase gene family in common bean are still unexplored. In the study, 29 PvLAC genes were identified through a whole-genome analysis of laccase genes in common bean. A comprehensive analysis of evolutionary relationships and stress expression profiles emphasizes the significant role of PvLAC genes in the response of common bean to abiotic stresses. The regulation of *PvLAC3*, *PvLAC4*, *PvLAC7*, and *PvLAC14* in response to cold and nicosulfuron stress was also explored, as well as potential inhibition in common bean growth and development, to lay the foundation of biological function research of common bean PvLAC gene family and the breeding of stress-resistant varieties.

## Materials and methods

### Identification of the laccase gene family members in common beans (*Phaseolus vulgaris*)

Genomic information for common bean (*Phaseolus vulgaris* v2.1) was retrieved from the Phytozome13 database (https://phytozome.jgi.doe.gov) (accessed at 2023.5.1). 17 laccase sequences from Arabidopsis were sourced from The Arabidopsis Information Resource (TAIR) database (http://arabidopsis.org) (accessed at 2023.5.1). Hidden Markov Model (HMM) profiles for Arabidopsis laccase conserved domains (PF00394, PF07731, PF07732) were obtained from the Pfam database (http://pfam.xfam.org/)(accessed at 2023.5.1). The domains were identified in the common bean *Proteome* using HAMMER 3.0. Outcomes from three BLAST searches were combined, and duplicates were removed, and sequences lacking laccase domains were excluded using default settings of the NCBI Conserved Domain Database (CDD) (https://www.ncbi.nlm.nih.gov/Structure/bwrpsb/bwrpsb.cgi) (accessed at 2023.5.1). The identified candidate genes underwent analysis for physical and chemical properties such as molecular weight (MW), isoelectric point (PI), sequence length, instability index, hydropathy, and three-dimensional structure prediction using ExPASy (https://www.expasy.org/)(accessed at 2023.5.1). Subcellular localization of the proteins was predicted using WoLF PSORT (https://wolfpsort.hgc.jp/)(accessed at 2023.5.1). The nomenclature of the PvLAC genes was based on the chromosomal distribution sequence.

### Phylogenetic analysis of PvLAC gene family members, collinearity analysis and gene structure analysis of the PvLAC gene family

Genome files, genome annotation files (gff3), and protein sequence files of Arabidopsis, common bean, and soybean were obtained from the Phytozome13 database. A total of 46 protein sequences from Arabidopsis (17) and common bean (29) were aligned using MEGA11, and a phylogenetic tree was constructed using the Maximum Likelihood (ML) method with 1000 replicates at all conserved sites. The phylogenetic tree was visualized and edited with ChiPlot (https://www.chiplot.online/)(accessed at 2023.6.10) [39]. A species evolutionary tree including Arabidopsis, common bean, and soybean was generated using timetree (http://www.timetree.org/)(accessed at 2023.7.23), with branch length and node information removed using MEGA11. The *LAC* family species evolutionary tree was constructed and analyzed using protein sequences and the species evolutionary tree of Arabidopsis (17), common bean (29), and soybean (93), with the Gene Gain & Lost Analysis plugin in TBtools [40]. Intra-species collinearity was examined using TBtools’ One Step MCScanX-Super Fast plugin and visualized with Advanced Circos. Inter-species collinearity of *LACs-s* among Arabidopsis, common bean, and soybean was depicted using the Multiple Synteny Plot. The ratio of non-synonymous to synonymous substitutions (Ka/Ks) at non-synonymous sites was determined using the Simple KaKs_Calculator. Conservative motifs in the laccase protein sequences from Arabidopsis and common bean were identified using the MEME online tool (http://meme-suite.org/tools/meme) (accessed at 2023.5.2). Additionally, the 2000 bp upstream promoter sequences of LAC genes from Arabidopsis and common bean were analyzed for *cis*-acting elements via the PlantCARE website (http://bioinformatics.psb.ugent.be/webtools/plantcare/html) (accessed at 2023.5.2) and the PlantPAN4.0 [41] (http://plantpan.itps.ncku.edu.tw/plantpan4/promoter_analysis.php) (accessed at 2024.6.25). Finally, TBtools was used to comprehensively present the phylogenetic tree, conservative motifs, gene structures, and *cis*-acting elements.

### Temporal and spatial expression patterns of the common bean LAC gene expression

RNA sequencing (RNA-Seq) data (based on the FPKM values) for common bean across 10 distinct tissues or developmental stages were obtained from the Phytozome13 database (https://phytozome.jgi.doe.gov) (accessed at 2023.7.1). Data visualization was conducted using TBtools, with the mature stage data being presented in a cartoon heatmap format through TBtools’ Fancy Heatmap Browser.

### Expression profiles of *PvLACs* in common bean response to cold and salt stress

The transcriptome data of common bean seedlings subjected to low temperature stress (GSE192891) was derived from the NCBI database (accessed at 2023.7.30), which provided gene expression data based on FPKM. The cold-tolerant and sensitive common bean seedlings were subjected to low temperature (4 °C) for 3 days [42]. The common bean salt stress transcriptome data (GSE156113) was derived from the NCBI database (accessed on 2023.7.18), providing files in SRA format. We used the Convert SRA to Fastq Files plug-in of TBtools to convert SRA format files into Fastq format files. By filtering the original data to delete the reads containing connectors and low-quality reads, high-quality clean reads could be obtained. Sequence alignment was performed using HISAT 2.0 software (reference genome: *Phaseolus vulgaris v2.1*), and gene expression was estimated using stringtie to obtain FPKM-based gene expression data. Two common bean varieties were selected in the experiment, and the seedlings were treated with NaCl (final concentration of 125 mM) for 3 days by hydroponics [34].

Excel was used to find and process the expression data of 29 *PvLACs-s*, and heatmaps was built through TBtools.

### Plant materials, VIGS plasmid vector and experiment strains

The common bean ' milk flower ' was purchased from Huajian Pastoral Seed Store (Songyuan, Jilin, China) and planted in the plant culture room of Heilongjiang Bayi Agricultural University (Daqing, Heilongjiang, China). The VIGS plasmid vectors pTRV1 and PNC-TRV2(pTRV2) were provided by NC Biotech, and the recombinant plasmids were constructed by seamless cloning. Competent Escherichia coli DH5α and Agrobacterium tumefaciens GV3101 were purchased from Tianyu Biotechnology.

### Treatment with exogenous hormones or low-temperature

Phaseolus vulgaris ' milk flower ' beans, a common variety, was used as experimental material. Experiments were conducted using potted plants in plastic pots (13.5 cm in diameter and 12 cm in height). The mix soil consisted of nutrient soil to vermiculite (4 mm in diameter) at 3:1 ratio. When the first true leaf fully expanded, three uniformly growing seedlings were selected to be treated.

Six distinct treatment groups were included in the experiment: CK (control), LT (low temperature), ABA (abscisic acid), ABA + LT, GA (gibberellic acid), and GA + LT, with three biological replicates. Treatments spraying with ABA (100 µM) and GA (100 µM) were evenly applied at 8:00 AM, while CK and LT groups treated with distilled water as controls at the same time. The low-temperature groups were subjected to cold stress in a plant incubator at 4 °C for 24 h, while the other groups were maintained at normal room temperature as controls [34, 43]. Samples of the first trifoliate leaf were collected at 0, 3, 6, 12, and 24 h after treatment. The phenotypic changes were photographed and physiological samples were taken to assess physiological indicators at 24 h exposed to cold stress.

### Virus-induced gene silenced plants construction and treatments with Nicosulfuron and cold stress

Phaseolus vulgaris ‘milk flower’ beans, a common variety, was used as experimental material. The SGN-VIGS Tool (https://vigs.solgenomics.net/) (accessed at 2023.8.10) was used to identify silencing fragments for *PvLAC3*, *PvLAC4*, *PvLAC7*, and *PvLAC14*. The TRV1 and TRV2 vectors were sourced from NC Biotech(ncloning.com), and the construction of pTRV2-*PvLAC3*, pTRV2-*PvLAC4*, pTRV2-*PvLAC7*, and pTRV2-*PvLAC14* vectors was performed using the Nimble Cloning kit (NC001) and pNC-AEnTopo blunt-end cloning kit (NC007) from NC Biotech. Transformation of the pTRV1, pTRV2, pTRV2-*PvLAC3*, pTRV2-*PvLAC4*, pTRV2-*PvLAC7*, and pTRV2-*PvLAC14* vectors into Agrobacterium tumefaciens strain GV3101 was conducted via heat shock [44, 45]. To simulate field soil contamination with nicosulfuron, a toxic soil method was employed. Nicosulfuron was incrementally mixed into the soil multiple times at a rate of 60 g/ha, ensuring uniform distribution [46]. Plants were grown in a growth chamber under standard conditions. Upon the emergence of true leaves, plants were subjected to weekly foliar and root treatments with Agrobacterium tumefaciens for four weeks. The Agrobacterium tumefaciens solution, consisting of equal quantities of pTRV1 and pTRV2 with an optical density between 4 and 6, was prepared and kept in dark conditions for three hours. In the fifth week, leaf sampling was conducted for qRT-PCR analysis to identify targeting gene silenced plants for further study. In the nicosulfuron experiment, samples were taken for molecular and physiological analyses. In the low-temperature experiment, half of the target gene silenced plants and TRV2 control plants were underwent cold stress at 4°C [34]in a plant growth chamber for 6d, followed by sample collection for molecular and physiological analysis.

### RNA extraction and qRT-PCR assays

RNA extraction was performed using Trizol reagent (TaKaRa, Dalian, China) according to the manufacturer’s instructions. RNA quality was assessed via gel electrophoresis and an Agilent 2100 bioanalyzer. First-strand cDNA synthesis was conducted using TransScript reverse transcriptase (TaKaRa, Dalian, China) following the manufacturer’s guidelines. The β-actin 11 gene of common bean served as the reference gene [47]. Gene-specific primers for qRT-PCR analysis were listed in ‘Supplementary Material.doc’. qRT-PCR experiments were carried out using the Applied Biosystems StepOnePlus real-time PCR system (Thermo Fisher Scientific). Each reaction mixture, totaling 10 µL, included 5 µL of 2×PerfectStart Green qPCR SuperMix, 0.5 µL of forward primer, 0.5 µL of reverse primer, 1 µL of cDNA, and 3 µL of RNase-free water. The thermal cycling program started with an initial denaturation at 95°C for 30 s, followed by 40 cycles of 95°C for 5 s and 60°C for 30 s. After each reaction, a melt curve analysis was conducted to confirm the specificity of the PCR products. Relative gene expression levels were determined using the 2^−ΔΔCt^ method.

### Measurement of physiological indicators and statistical analysis

The content of MDA was determined by thiobarbituric acid method, and the activity of SOD was determined by NBT photoreduction method. POD activity was measured by guaiacol method, and the activity of CAT and APX was measured by ultraviolet spectrophotometry [48]. The chlorophyll content was determined by 95% ethanol extraction colorimetry. The main veins of the leaves were removed and 0.1 g fresh samples were added with 10 mL of 95% ethanol solution in a 25 mL scale tube. The solution was extracted overnight in the dark until the tissue was completely bleached; the extract was diluted to 15 mL with 95% ethanol solution. The absorbance was measured at wavelengths of 665 nm, 649 nm and 470 nm using an ultraviolet spectrophotometer.

Statistical analyses were conducted using ANOVA via SPSS software, with mean comparisons made using Duncan’s t-test at a 5% significance level. Data visualization was performed using Origin 8.0 (MicroCal Inc, Northampton, MA, USA).

## Results

### Identification of the *PvLAC* gene family in common bean

Following to the framework of the Arabidopsis laccase gene family, 29 laccase genes were identified and named *PvLAC1* to *PvLAC29* according to the genomic location. Notably, *PvLAC29* is derived from the extranuclear genome. The physicochemical properties of 29 laccase proteins were comprehensively analyzed, and subcellular localization was predicted (Table S1). *PvLAC20* was the longest at 610 amino acids and the heaviest at 67.16 kDa, while *PvLAC1* was the shortest at 219 amino acids and the lightest at 23.60 kDa. The isoelectric point (PI) values ranged from 6.42 (*PvLAC25*) to 10.06 (*PvLAC18*), with the majority (86.21%) displaying a PI greater than 7, indicating a predominance of alkaline amino acids [49]. The instability coefficients of all 29 laccase proteins were below 40, indicating the stability [50]. The subcellular location of 29 laccase proteins was predicted, and 19 laccase proteins were located in the chloroplast, while 4 proteins were located in the cytoplasm, and 3 proteins were located in the vacuole, and 1 laccase protein was located in the peroxisome, cell wall and mitochondria. Additionally, structural predictions revealed that two proteins (*PvLAC18* and *PvLAC29*) among the 29 featured 2 transmembrane domains (Figure S1).

### Systematic evolutionary analysis of the laccase gene family in common bean

**Fig. 1:**
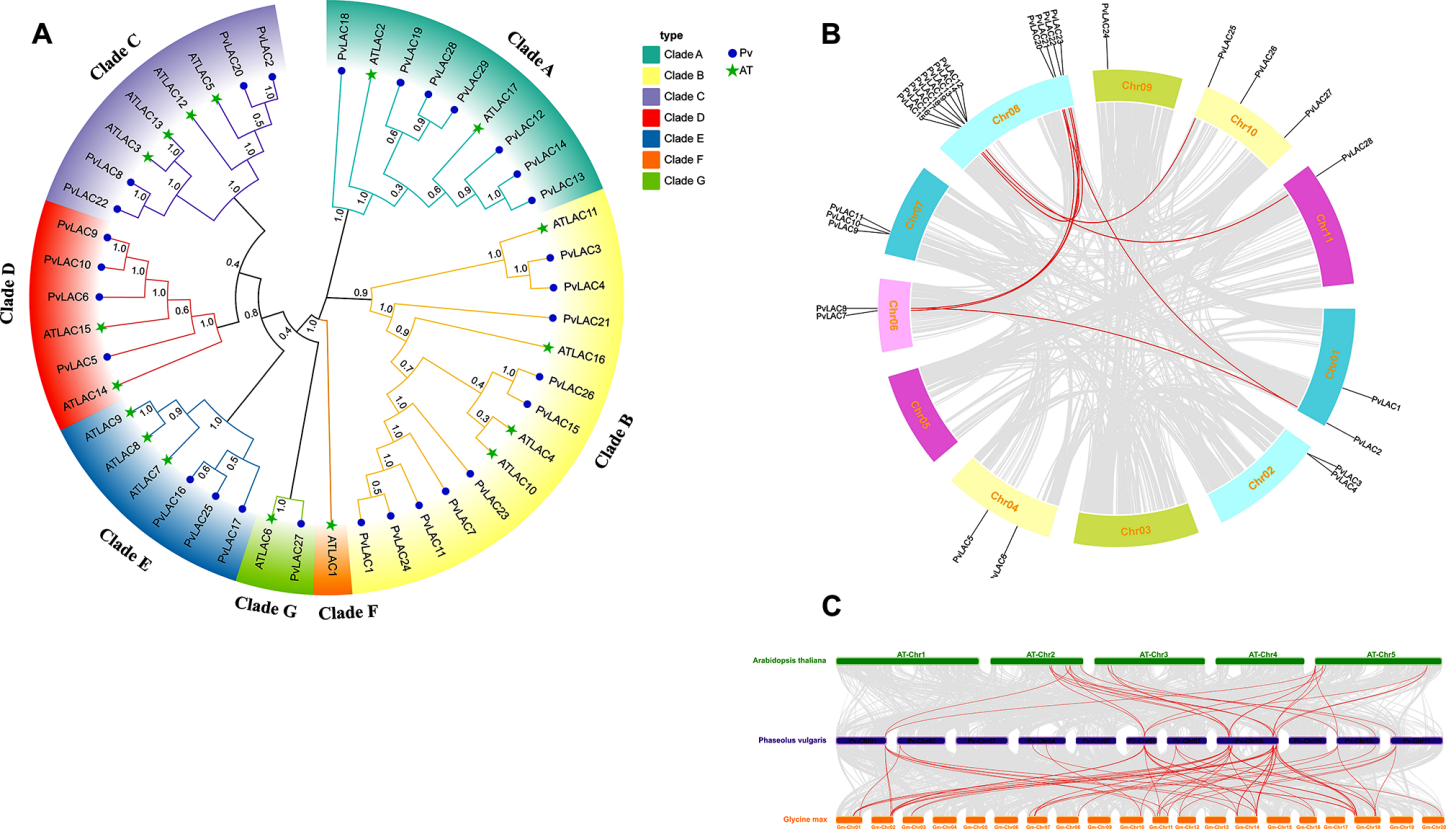
Phylogenetic analysis and synteny analysis of the LAC genes. **(A)** The phylogenetic tree was constructed using 17 protein sequences of Arabidopsis and 29 protein sequences of common beans. The gene family was divided into 7 subfamilies, each distinguished by a unique color. Green pentagrams and purple circles represent LAC genes in Arabidopsis and common beans, respectively. **(B)** A synteny analysis of the PvLAC gene family was conducted. All PvLAC genes were located on chromosomes, with pairs of identified PvLAC genes highlighted in red and interconnected by red lines. **(C)** A synteny analysis of LAC genes was performed in Arabidopsis, common beans, and soybeans. The chromosomes of each species were depicted in different colors, and the homologous gene pairs were illustrated by red lines

The evolutionary relationship between common bean and Arabidopsis laccase was elucidated by phylogenetic analysis. A phylogenetic tree consisting of 7 different subclades centered on *AtLACs-s* was constructed with the protein sequences of 29 *PvLACs-s* and 17 *AtLACs-s*. The 29 *PvLACs-s* were distributed in 6 subclades, each containing a different number of members (Fig. 1A). Each subclades exhibited a unique composition: subclades A included 7 *PvLACs-s* and 2 *AtLACs-s*, subclades B comprised 10 *PvLACs-s* and 4 *AtLACs-s*, subclades C was consisted of 4 *PvLACs-s* and 4 *AtLACs-s*, while subclades D, E, and G contained 4, 3, and 1 *PvLACs-s*, and 2, 3, and 1 *AtLACs-s*, respectively. The classification indicated that the common bean laccase gene family had a replication event during evolution. The expansion and contraction of the LAC gene family in the evolution of Arabidopsis, soybean and common bean species were revealed. The LAC gene family of common beans was significantly contracted, while the soybean LAC gene family was significantly expanded (Figure S2).

In order to elucidate the evolutionary impact of the past, the genomic distribution and synteny of 29 *PvLACs-s* on chromosomes were analyzed. An uneven distribution was observed, with chromosome 8 with the most (12) and chromosome 9 with the least (1) *PvLACs-s* (Fig. 1B). Eleven pairs of synteny genes were identified, and Ka / Ks was less than 1, indicating that purification selection affected the evolution of the laccase gene family (Table S2). Additionally, we constructed an interspecies synteny map of Arabidopsis, common beans, and soybeans, highlighting a stronger genetic relationship between soybeans and common beans compared to that between Arabidopsis and common beans. Specifically, 55 pairs of synteny genes were identified between common bean and soybean, while only 22 pairs of synteny genes were identified between Arabidopsis and common bean (Fig. 1C).

### Gene structure and *cis*-acting element analysis of the PvLAC gene family members

**Fig. 2:**
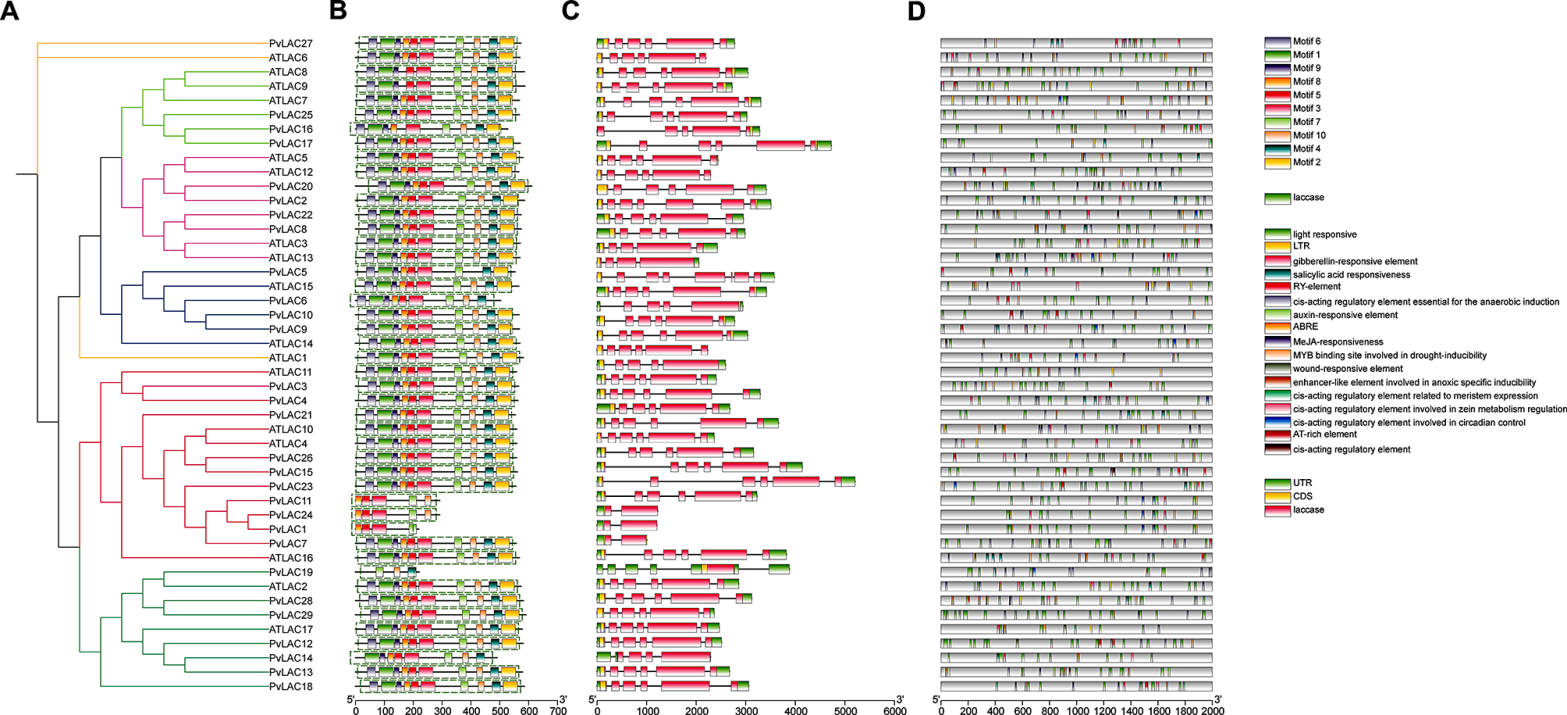
Phylogenetic tree, conserved motifs, gene structure, and *cis*-acting elements in the promoter region of *LACs-s* in Arabidopsis and common bean. **(A)** Phylogenetic relationship of *AtLACs-s* and *PvLACs-s*. **(B)** Conserved motifs and the distribution. Conserved motifs were identified in the top right corner and displayed in various colors. The structure enclosed by the green box represents the laccase structural domain. **(C)** Gene structure of *LACs-s*. UTR, CDS, laccase structural domain, and introns were represented by green boxes, yellow boxes, pink boxes, and grey lines, respectively. **(D) ***Cis*-acting elements in the promoter region of *LACs-s*. Different colors represent various elements

To further understand the structure of *LACs*-s protein, the conserved motifs of *LACs*-s in Arabidopsis and common beans were predicted, and the gene structure and *cis*-acting elements were analyzed. The results showed that the LAC family was highly conserved because the number and type of conserved motifs were relatively consistent among different subfamily members (Fig. 2A Fig. 2B). The LAC domain of most PvLAC genes was divided into 5 fragments by introns, and the arrangement patterns within each subfamily were consistent (Fig. 2B Fig. 2C). Despite the structural similarity of *LACs-s* in the same subfamily, the structure of *PvLAC1*, *PvLAC11*, *PvLAC19* and *PvLAC24* was observed to be lost although the complete laccase domain was retained. The structural deletions of *PvLAC1*, *PvLAC11*, *PvLAC19* and *PvLAC24* might be due to sequencing errors or the discarding of redundant parts, which only retain the basic core structure in evolution. In addition, these genes might be pseudogenes.

In order to further explore the potential regulatory pathways of PvLAC genes, the upstream promoter sequences (2000 bp) of Arabidopsis and common bean *LACs-s* were searched through PlantCARE and PlantPAN4.0 (Fig. 2D Figure S3 Figure S4). A wide range of core cis-acting elements was identified and categorized into 17 functional groups, including light and hormone response elements. *PvLACs-s* could be regulated by light signals because a large number of light-responsive elements had been detected in the promoter region. Stress response elements and various hormone response elements were also been found, including low temperature, abscisic acid, gibberellin, methyl jasmonate, salicylic acid, and auxin. Among the 29 *PvLACs-s*, low temperature response elements were detected in 8 *PvLACs-s*, while gibberellin response elements were identified in 17 *PvLACs-s*, and abscisic acid response elements were explored in 20 *PvLACs-s*. In addition, 14 *PvLACs-s* possessed MYB binding sites linked to drought induction, highlighting a potential role in drought and other stresses response. Through PlantPAN 4.0 search, PvLAC genes were found to be regulated by potential transcription factors, including *B3*, *NAC*, *TCP*, *WRKY*, *MYB*, *Dof* and other transcription factor families involved in plant response to stresses, which indicated that the PvLAC gene family has a potential regulatory role in various stresses (Figure S4).

### The tissue-specific expression patterns of *PvLACs-s* and the induction of PvLAC gene expression by ABA and GA

**Fig. 3:**
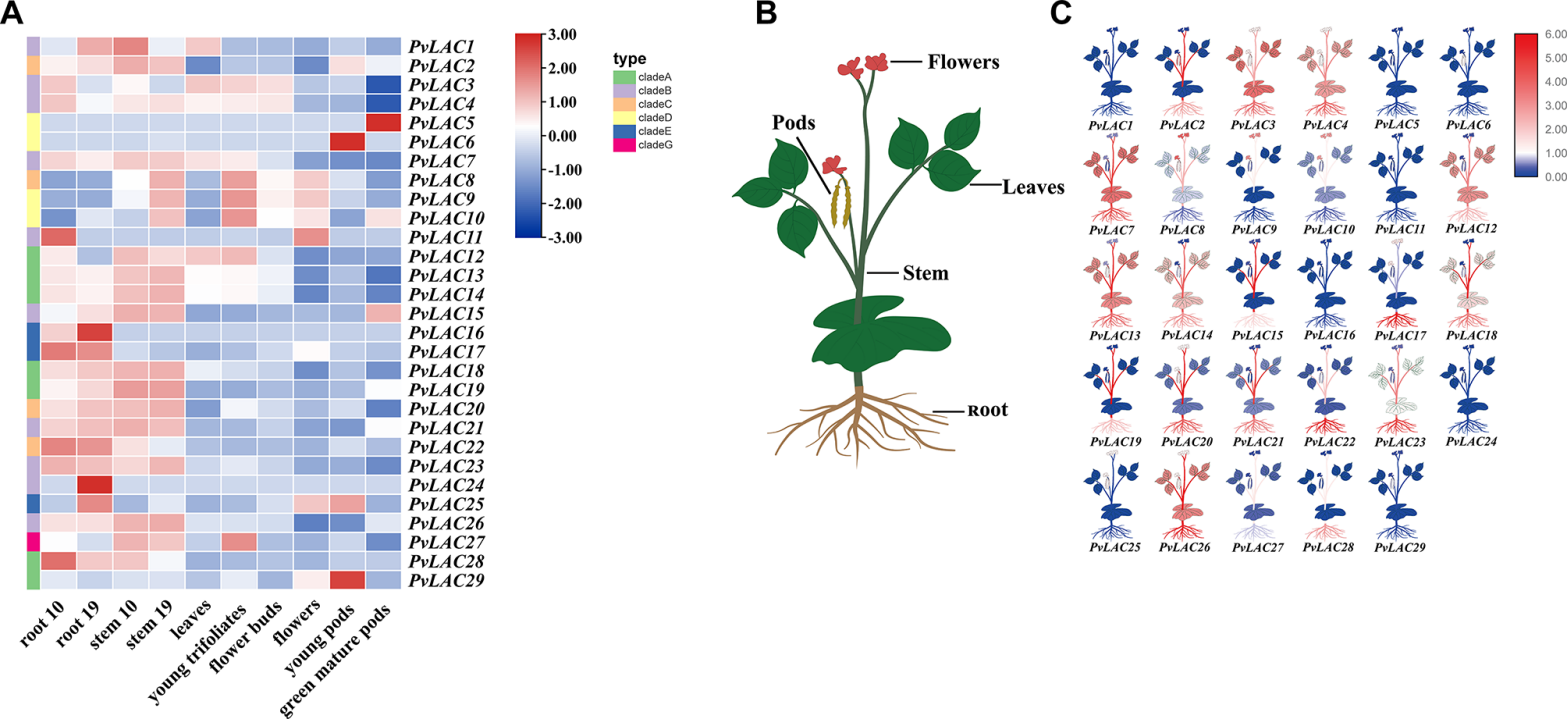
Expression patterns of PvLAC gene in different tissues/organs and development. **(A)** Expression heatmaps of PvLAC genes in different tissues/organs and during development. **(B)** Cartoon illustration of a common bean. **(C)** Expression level of *PvLACs-s* in various tissues/organs

The tissue-specific expression patterns of the PvLAC gene in various developmental stages of common beans were analyzed (Fig. 3). Diverse expression levels were displayed by each PvLAC gene within the same tissue types, particularly in roots and stems. Furthermore, unique transcript abundance was observed for each gene across different tissues and temporal stages. Generally, *PvLACs-s* were predominantly expressed in roots and stems, suggesting the vital role in the growth and function of these structures. Specifically, nine genes (*PvLAC3*, *PvLAC4*, *PvLAC7*, *PvLAC12*, *PvLAC13*, *PvLAC14*, *PvLAC18*, *PvLAC23*, and *PvLAC26*) were expressed in leaves but exhibited higher expression levels in roots and stems. Conversely, seven genes (*PvLAC1*, *PvLAC5*, *PvLAC6*, *PvLAC11*, *PvLAC16*, *PvLAC24*, and *PvLAC29*) showed low expression levels across all tissues, with *PvLAC1*, *PvLAC11*, and *PvLAC24* exhibiting deficient gene structures. The distinctive expression patterns of *PvLACs-s* in terms of time and location suggested a correlation with the specific functions.

**Fig. 4:**
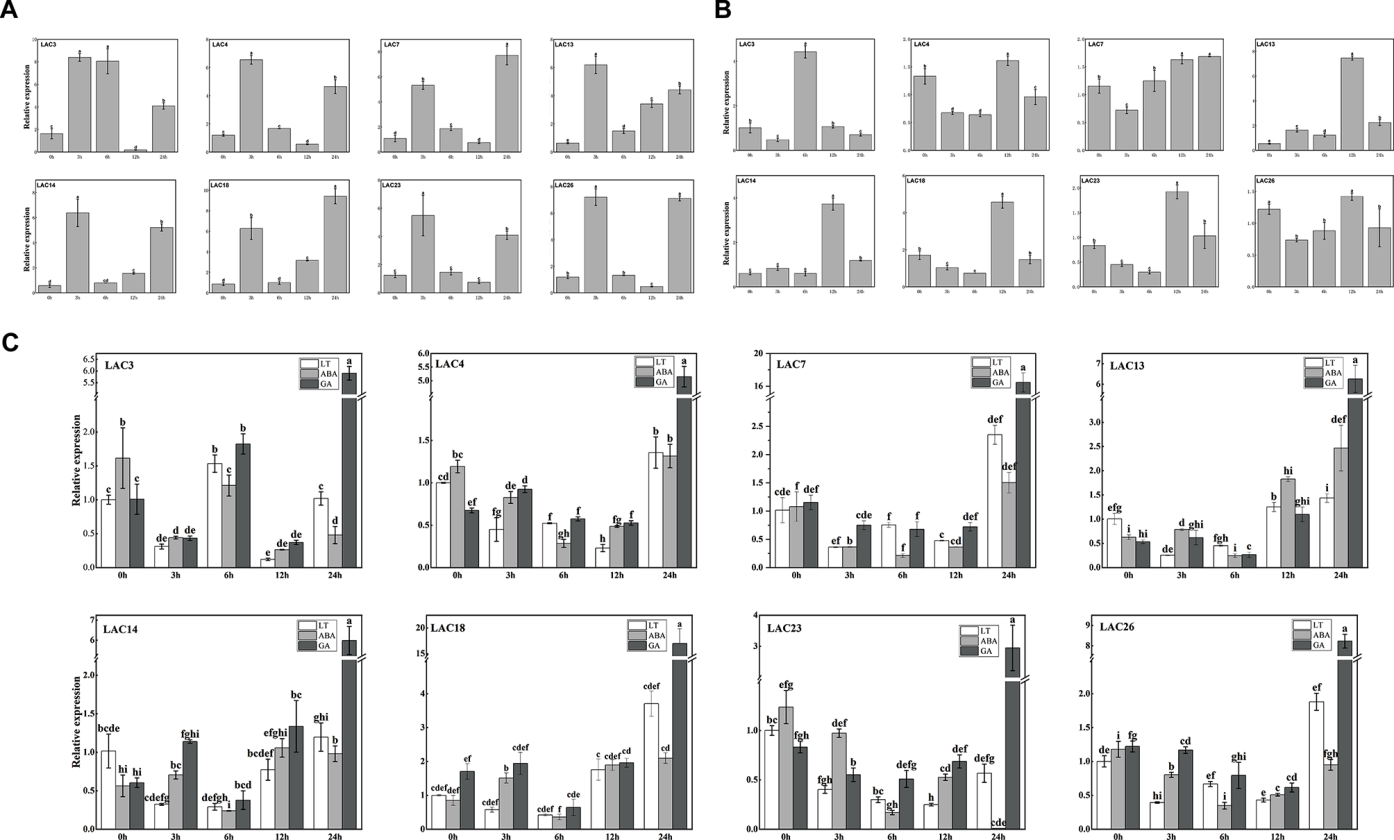
Expression pattern of PvLAC genes under different treatments. According to Duncan’s multiple tests, columns with different letters indicate significant differences (*p* < 0.05). **(A)** Expression profiles of PvLAC genes under ABA treatment. **(B)** Expression profiles of PvLAC genes under GA treatment. **(C)** Expression profiles of PvLAC genes under low-temperature stress induced by ABA and GA

In response to environmental stress, plants activate specific defense mechanisms mediated by several signaling pathways, involving in ABA, GA, ethylene, and jasmonic acid. Following the analysis of *cis*-acting elements in the promoter region, we treated common bean leaves with abscisic acid and gibberellic acid. Using qRT-PCR, the expression of eight genes (*PvLAC3*, *PvLAC4*, *PvLAC7*, *PvLAC12*, *PvLAC13*, *PvLAC14*, *PvLAC18*, *PvLAC23*, and *PvLAC26*) at 0, 3, 6, 12, and 24-hour intervals at room temperature were monitored. The results indicated that both ABA and GA significantly induced the expression of above genes, with peaks varying between genes and hormones (Fig. 4A and B). Additionally, under low-temperature conditions, gene induction by ABA and GA differed significantly from that at room temperature, with GA induced the maximum expression levels at 24 h, and the expression patterns of these genes at low temperatures induced by ABA displaying considerable variability (Fig. 4C). The evidence suggested that ABA and GA could differentially modulate the response of *PvLACs-s* to low-temperature stress, likely reflecting the varied functional roles of these genes.

### The expression of PvLAC gene family members under cold and salt stress

**Fig. 5:**
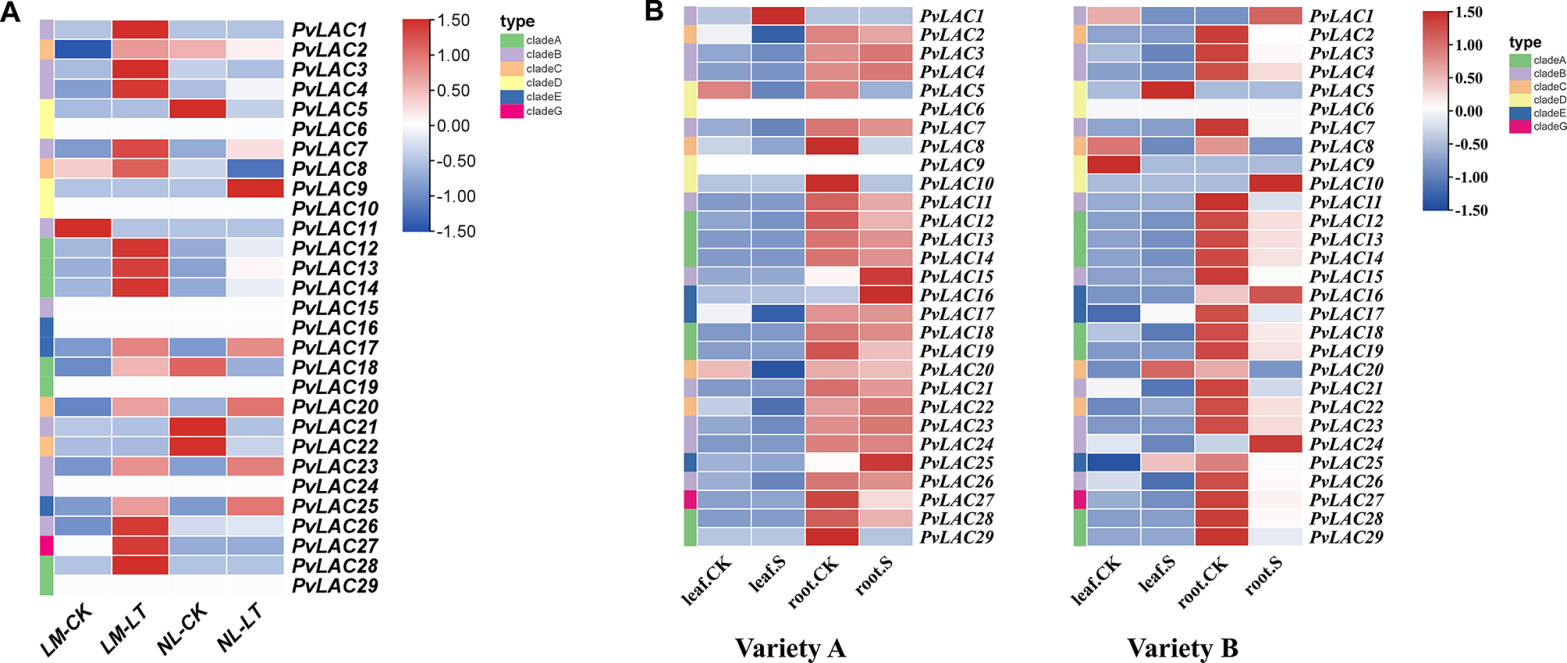
Heatmap of PvLAC gene expression under low temperature and salt stress. Blank spaces indicated missing data. **(A)** The expression level of PvLAC gene in two common varieties (cold sensitive (LM) and cold tolerant (NL)) under the same low temperature treatment, LT represented low temperature treatment. **(B)** Heatmap of PvLAC gene expression in roots and leaves of common bean varieties A and B under salt stress, where S represented salt treatment

Using transcriptome data sourced from the NCBI database, the expression heatmaps of the common bean laccase gene family in response to low temperature and salt stress was analyzed. A significant number of laccase genes were notably upregulated in cold-sensitive varieties under low temperature conditions, while a subset in cold-tolerant varieties also exhibited increased expression (Fig. 5A). These observations indicated that laccases could play a crucial role in the low-temperature response of common beans, potentially offering a beneficial effect. Conversely, under salt stress, the inhibition of the majority of laccase gene expressions was exhibited in the roots of variety B. However, a small number of genes were highly induced, possibly contributing to the adaptation of roots to salt stress. In variety A, a similar pattern in the expression trend of laccase genes was observed, though with less variation (Fig. 5B). Under salt stress conditions, only a few genes showing significantly induced expression was likely to have predominant function of laccase genes within the roots.

### The function verification of *PvLACs-s* under different stresses by Virus-induced gene silence (VIGS)

#### The function of *PvLAC3*, *PvLAC4*, *PvLAC7* and *PvLAC14* under nicosulfuron stress

**Fig. 6:**
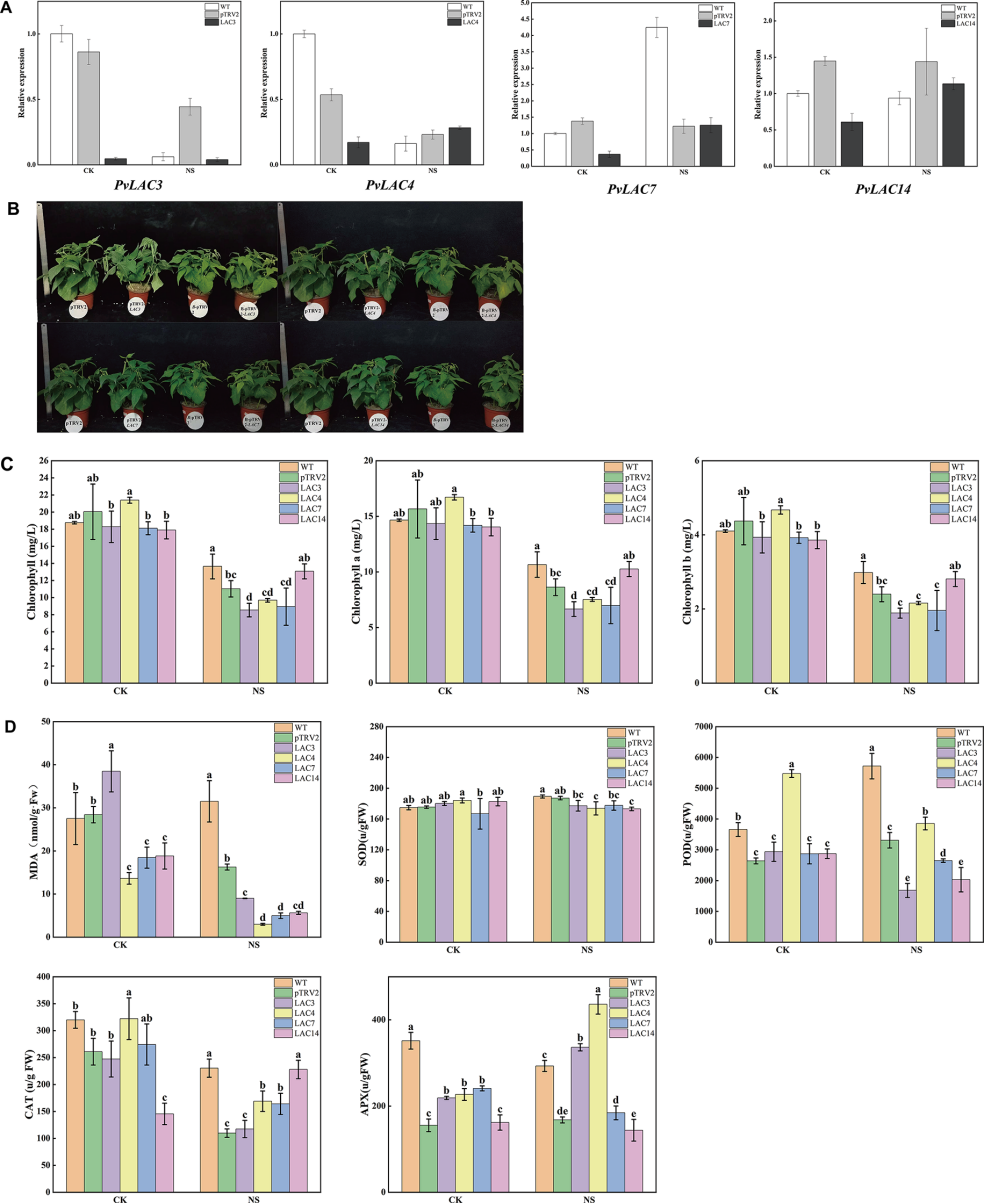
*PvLAC3*, *PvLAC4*, *PvLAC7*, and *PvLAC14* were involved in the regulation of nicosulfuron stress. **(A)** Gene silencing efficiency through qRT-PCR, where NS represented nicosulfuron treatment, WT indicated the wild type of group, and pTRV2 was the control group. **(B)** The phenotype of *PvLAC3*, *PvLAC4*, *PvLAC7*, and *PvLAC14* silenced plants under nicosulfuron stress, where B represented nicosulfuron treatment. **(C)** Chlorophyll content in plants under nicosulfuron stress, where NS represented nicosulfuron treatment. **(D)** MDA content and activities of four antioxidant enzymes (SOD, POD, CAT, APX) in each treatment, where NS represented nicosulfuron treatment. MDA: malonaldehyde; SOD: superoxide dismutase; POD: peroxidase; CAT: Catalase; APX: ascorbate peroxidase

To elucidate the role of laccases in the response of common beans to herbicides, an increase in expression of eight genes (*PvLAC3*, *PvLAC4*, *PvLAC7*, *PvLAC13*, *PvLAC14*, *PvLAC18*, *PvLAC23*, *PvLAC26*) with exposure to nicosulfuron was initially confirmed, as determined by quantitative real-time polymerase chain reaction (qRT-PCR) analysis (Figure S5). Subsequently, we employed virus-induced gene silence (VIGS) to inhibit the expression of *PvLAC3*, *PvLAC4*, *PvLAC7* and *PvLAC14* in common beans. Compared to pTRV2 control plants, the transcription level of these genes exhibited reduction ranging from 57.9 to 94.6%. Notably, under nicosulfuron treatment, only *PvLAC3* silenced plants showed a significant reduction in gene expression in silenced plants compared to pTRV2 plants (Fig. 6A).

The observation of phenotype (Fig. 6B) revealed that silence of *PvLAC3*, *PvLAC4*, *PvLAC7* and *PvLAC14* gene induced podding earlier under standard growth conditions. Moreover, these silenced plants also podded earlier than pTRV2 plants when exposed to nicosulfuron treatment, and the leaves turned yellow. The chlorophyll content exhibited a decrease in *PvLAC3*, *PvLAC4* and *PvLAC7* silenced plants compared to pTRV2 plants, while *PvLAC14* silenced plants showed a higher level (Fig. 6C).

The evaluation of malondialdehyde (MDA) content revealed that silenced plants under nicosulfuron treatment exhibited lower MDA level, compared to pTRV2 plants. Additionally, the peroxidase (POD) activity in *PvLAC3*, *PvLAC7* and *PvLAC14* silenced plants was lower than pTRV2 plants, while catalase (CAT) activity in *PvLAC4*, *PvLAC7*, and *PvLAC14* silenced plants was higher than pTRV2 plants, although the superoxide dismutase (SOD) activity in silenced plants did not change significantly. These findings suggested that these genes might play diverse roles in antioxidant regulation (Fig. 6D).

In summary, the resistance of common beans to nicosulfuron was reduced by silencing *PvLAC3*, *PvLAC4* and *PvLAC7*, while the inhibition of *PvLAC14* enhanced the resistance of common beans to nicosulfuron. It is worth noting that the development of common bean seems to be accelerated by the silence of *PvLAC3*, *PvLAC4*, *PvLAC7* and *PvLAC14* gene.

#### The function of *PvLAC3*, *PvLAC4*, *PvLAC7* and *PvLAC14* under cold stress

**Fig. 7:**
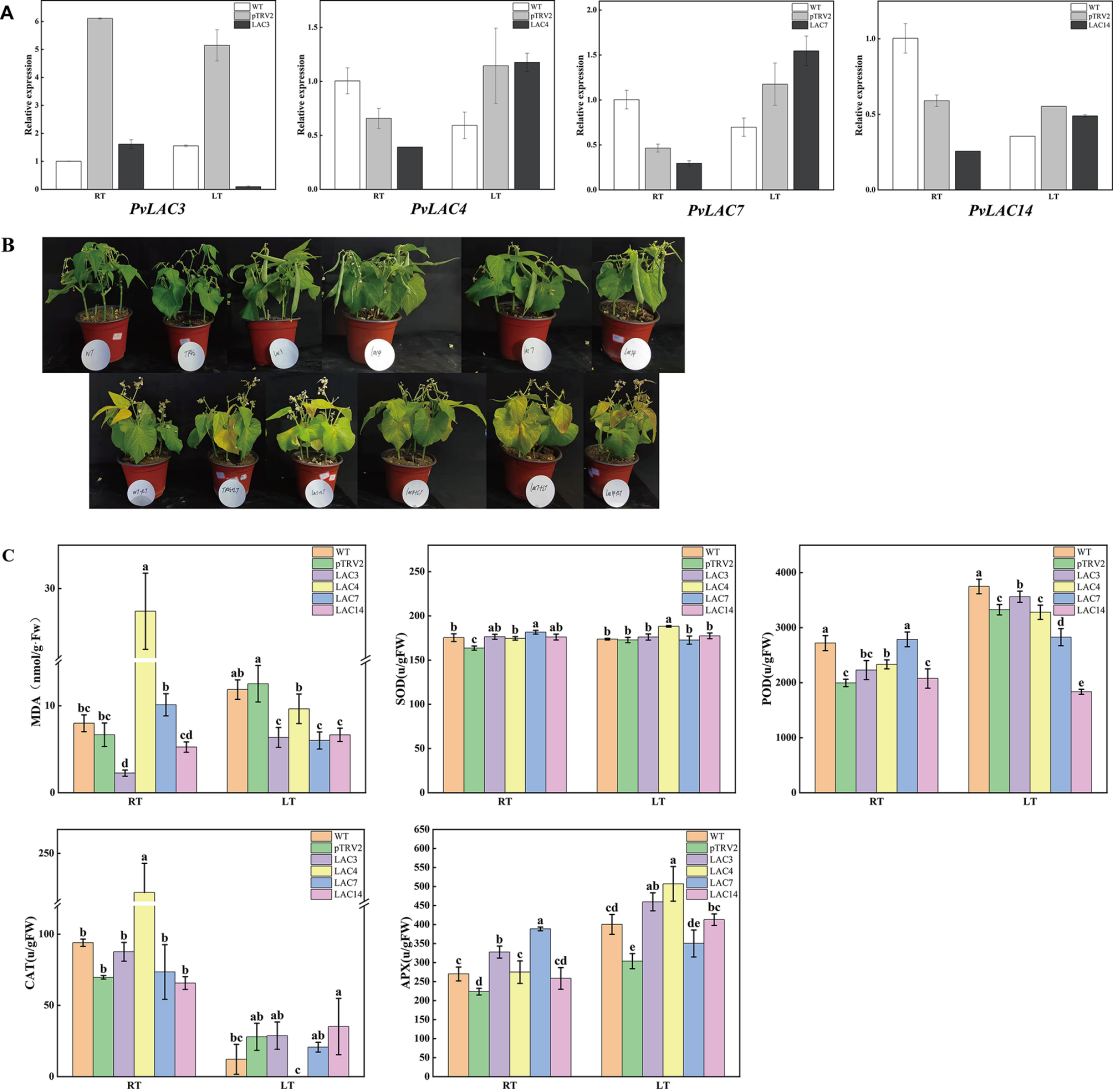
Regulation of *PvLAC3*, *PvLAC4*, *PvLAC7*, *PvLAC14* on cold stress. **(A)** Gene silencing efficiency through qRT-PCR, LT: treatment of 4 °C cold temperature, RT: room temperature, WT: wild type, pTRV2: control group. **(B)** After being subjected to cold stress, the phenotypes of *PvLAC3*, *PvLAC4*, *PvLAC7*, and *PvLAC14* silenced plants were restored to room temperature for 3 days. **(C)** MDA content and activities of four antioxidant enzymes (SOD, POD, CAT, APX) in each treatment. MDA: malonaldehyde; SOD: superoxide dismutase; POD: peroxidase; CAT: Catalase; APX: ascorbate peroxidase

In order to explore the role of laccase in soybean cold resistance, the expression of *PvLAC3*, *PvLAC4*, *PvLAC7* and *PvLAC14* genes in common beans was inhibited by virus-induced gene silencing. The transcription level of these genes decreased, ranging from 36.4 to 73.6%, compared to the pTRV2 control plants. A significantly lower gene expression was observed in *PvLAC3*-silenced plants, while *PvLAC14*-silenced plants exhibited only a minor decrease after cold stress (Fig. 7A).

Under room temperature conditions, the gene-silenced plants demonstrated a more rapid pod-setting tendency. With exposure to low temperature, these plants displayed more wilting leaves compared to pTRV2 plants (Figure S6). The growth and development of common beans were substantially inhibited under cold stress, while the development of gene-silenced plants surpassed both the wild-type and pTRV2 plants. Notably, severe rust symptoms were observed on the leaves of *PvLAC3*, *PvLAC7* and *PvLAC14* silenced plants after 3d with exposure to cold stress, highlighting the severity of cold-induced damage (Fig. 7B).

Physiological analyses indicated that *PvLAC3*, *PvLAC7* and *PvLAC14* silenced plants exhibited lower malondialdehyde (MDA) levels compared to *PvLAC4* silenced plants and pTRV2 plants under cold stress. Assessment of the activity of superoxide dismutase (SOD), peroxidase (POD), catalase (CAT) and ascorbate peroxidase (APX) in *PvLAC4* silenced plants revealed that SOD activity of PvLAC4 silenced plants surpassed that of other plants, while the CAT activity was suboptimal under low temperature (Fig. 7C). These observations indicated that an enhanced antioxidative response to cold stress was demonstrated by *PvLAC4* gene silence, thereby mitigating damage. In contrast, a reduced response to cold stress was exhibited by gene silence of *PvLAC3*, *PvLAC7* and *PvLAC14*, resulting in more severe damage during the recovery phase at room temperature.

In summary, the susceptibility of common beans to cold stress was increased with the loss of function of *PvLAC3*, *PvLAC7* and *PvLAC14*, while the effect of *PvLAC4* was the opposite. In addition, the silence of *PvLAC3*, *PvLAC4*, *PvLAC7* and *PvLAC14* accelerated the development of common beans.

## Discussion

Laccases, represented as the largest subfamily among multicopper oxidases, played a critical role in the biosynthesis of lignin, an essential component for plant cell morphology and the development of secondary cell walls [26, 51, 52]. Additionally, laccases contribute to plant defense mechanisms against a wide range of biotic and abiotic stresses [53]. To conduct a comprehensive analysis of the laccase gene family in common bean, 29 *PvLACs-s* were identified in the common bean genome. The number was lower compared to other species, such as *Glycine max* (93) [5], *Eucalyptus grandis* (53) [54], *Brassica napus* (45) [25], *Gossypium spp.* (44) [23] and *Populus trichocarpa* (49) [55], but exceeded that of *Arabidopsis thaliana* (17) [22], *moso bamboo* (23) [56], and *Citrus sinensis* (24) [57]. The expansion of laccase gene family in common bean was limited.

The *Arabidopsis thaliana* laccase gene family was classified into six subclades, providing a basis for further research [22]. However, our study discerned a clear separation between the *AtLAC6*, *AtLAC14* and *AtLAC15* subclades and the *AtLAC7*, *AtLAC8and AtLAC9*, resulting in the creation of an independent subclades containing *AtLAC6*. Consequently, we reclassified *Arabidopsis thaliana* into seven subclades, with *PvLACs-s* distributed across six of them. The number of genes increased in subclades A, B, and D, while the number was decreased in subclades F. Intra-specific homology analysis of *PvLACs-s* identified 11 pairs of synteny genes, indicating gene duplication within the common bean laccase gene family [58]. Through Ka / Ks analysis, the evolutionary trajectory of the laccase gene family was mainly influenced by purification selection [59]. Moreover, a synteny analysis of laccase gene protein sequences in common beans, soybeans, and *Arabidopsis thaliana* revealed an expansion of the gene family during species evolution. Different degree of duplication and loss among members of the laccase gene family had been elucidated and revealed through the phylogenetic tree and species evolution tree of the laccase gene family. During the whole genome duplication event in leguminous plants, 21 genes were duplicated, and 4 genes were lost. Subsequent duplication events showed that common beans duplicated 4 genes but lost 12, whereas soybeans duplicated 59 genes and lost only 3. The different evolutionary routes of laccase gene family between common beans and soybeans might be attributed to different stress [60].

Motifs, associated with biological functions, were conserved sequences that may contain specific binding sites or shared sequence fragments involved in certain biological processes [61]. Neighboring *PvLACs*-s usually had more similar gene structure and motif composition was revealed by phylogenetic tree. However, a lack of motifs was observed in some members of groups A and B, despite having a complete laccase core domain. These members might have acquired new functions during evolution, or they might be pseudogenes [62]. The gene loss of the laccase gene family in the evolutionary process was identified, which could be seen from the phylogenetic tree.

The expression level of *PvLACs-s* in roots and stems were found to be higher than those in other plant tissues through temporal and spatial expression analysis. The trend coincided with the lignification levels across different tissues of common bean plants and was closely associated with the function of laccase in the biosynthesis of lignin [63]. Remarkably, members of Group D showed lower expression in roots and stems, while *PvLAC5* and *PvLAC6* exhibited enhanced expression during fruit pod development, suggesting the potential prominence in the process of fruit pod ripening. A comprehensive review of all plant parts revealed that nine genes—*PvLAC3*, *PvLAC4*, *PvLAC7*, *PvLAC12*, *PvLAC13*, *PvLAC14*, *PvLAC18*, *PvLAC23*, and *PvLAC26*—were expressed at the highest level within the leaves. These genes might play alternative roles, need to be further investigated in subsequent research.

The expression of *PvLACs-s* was mainly regulated at the transcriptional level by *cis*-element analysis. However, existing literature also suggested that the control of laccase genes could occur at the post-transcriptional level or through post-translational modifications [64]. Specifically, the upregulation of the *AtLAC4* gene was demonstrated to be mediated by *MYB58’s* binding to the AC element [65]. Therefore, potential regulatory approaches had been found by analyzing the promoter region of *PvLACs-s*. The *cis*-acting elements in response to low temperature, abscisic acid, gibberellin, methyl jasmonate, salicylic acid and auxin were predicted, and it was found that PvLAC genes were potentially regulated by transcription factor families (*B3*, *NAC*, *Dof*, *Myb*, *WRKY*, *TCP*) related to the regulation of stress, indicating that *PvLACs*-s may play a role in regulating plant growth and development and resistance to biotic and abiotic stresses [24, 25, 43, 66–70]. In order to verify this effect, common beans were treated with ABA, GA and low temperature, and the response of laccase gene family under low temperature and salt stress was analyzed by NCBI transcriptome data. *PvLAC3*, *PvLAC4*, *PvLAC7*, *PvLAC13*, *PvLAC14*, *PvLAC18*, *PvLAC23*, and *PvLAC26*, which had the highest expression level in leaves, were regulated by ABA and GA induction, but ABA and GA exhibited distinct regulatory mechanisms under low temperature, possibly due to differences in the regulatory pathways at low temperature [71]. Most of the laccase genes in the roots were inhibited under salt stress, but a few laccase genes were induced by salt stress and highly expressed. A hypothesis was proposed that these select genes could serve as crucial regulators of lignin synthesis, thereby enhancing cell wall strength through lignin accumulation. Consequently, the absorption of salt ions and subsequent damage to cell morphology were reduced [72–74].

Most laccase genes were considered to contain cold response elements, and the response of laccase genes to cold stress *was* mainly positive. Therefore, *PvLAC3*, *PvLAC7*, *PvLAC4* and *PvLAC14* were studied because of higher expression level. Under low temperature, the positive regulation of *PvLAC3*, *PvLAC7* and *PvLAC14* was shown, while the negative regulation of *PvLAC4* was found. Under cold stress, the biosynthesis of plant secondary metabolites might be regulated by *PvLAC3*, *PvLAC7* and *PvLAC14*. Moreover, the antioxidant enzyme system was activated by the metabolites after exposure to cold stress, which might explain why *PvLAC3*, *PvLAC7* and *PvLAC14* silenced plants exhibited lower MDA content and reduced SOD enzyme activity after cold stress, compared to pTRV2 plants [53, 75]. Silence of *PvLAC4* gene might also lead to the accumulation of certain secondary metabolites to enhance the cold resistance of common beans, consistent with the results of Hu et al. [53].

Subsequently, the regulatory effects of *PvLAC3*, *PvLAC4*, *PvLAC7* and *PvLAC14* under nicosulfuron stress were examined. Damage induced by nicosulfuron in common beans primarily affected the morphology and growth of plant, as evidenced by leaf turned yellow and plant growth reduced due to nicosulfuron treatment. Compared with pTRV2, limited growth and lower chlorophyll content were shown in *PvLAC3*, *PvLAC4* and *PvLAC7* gene silenced plants, while better growth and higher chlorophyll content were found in *PvLAC14* gene silenced plants. Some laccase genes played an important role in detoxification, and *PvLAC3*, *PvLAC4* and *PvLAC7* were speculated to be involved in the detoxification process, which positively affected the response of common beans to nicosulfuron [27]. The production of certain secondary metabolites might be potentially stimulated by *PvLAC3*, *PvLAC4* and *PvLAC7*, thereby contributing to the response to nicosulfuron. Interestingly, we noted that plants with silenced *PvLAC3*, *PvLAC4*, *PvLAC7* and *PvLAC14* genes demonstrated faster growth and developmental rates, which resulted in an increase in pod numbers and larger pods compared to wild type and pTRV2 controls at the same time point. We speculated that the growth and development of common beans were inhibited by laccase due to the phenylpropanoid metabolic pathway [76]. However, further research is required to fully understand the inhibitory mechanism.

## Conclusions

The structural characteristics, phylogenetic relationship and expression patterns of 29 PvLAC genes were characterized and comprehensively analyzed. The evolutionary relationship of laccase gene family among different species (*Arabidopsis thaliana*, common bean, soybean) was elucidated. The potential roles of PvLAC genes in light signal transduction, abiotic stress responses, and hormone responses were uncovered through *cis*-acting element analysis. The PvLAC gene family was involved in abiotic stress and hormone response. The key regulatory roles of *PvLAC3*, *PvLAC4*, *PvLAC7* and *PvLAC14* in the response to low temperature and nicosulfuron stress were verified, as well as the potential inhibitory effect on the growth and development of common bean.

### Electronic supplementary material

Below is the link to the electronic supplementary material.

Supplementary Material 1

Supplementary Material 2

Supplementary Material 3

## Data Availability

The data supporting the findings of this study are available from the corresponding author (Yuxian Zhang), upon request.
