# Supplementary material for: Genome-wide analysis of the common bean (Phaseolus vulgaris) laccase gene family and its functions in response to abiotic stress
Source: BMC Plant Biol. 2024 Jul 19;24:688. doi: 10.1186/s12870-024-05385-x (PMC11264805; doi:10.1186/s12870-024-05385-x)
Supplement: Supplementary file 3 — Supplementary Material 3 [file 12870_2024_5385_MOESM3_ESM.doc]

**Attached table**.

**Table S1.** Information about members of the common bean gene family

**Table S2.** Ka/Ks (Ka: non-synonymous substitutions, Ks: synonymous substitutions)

| **Duplication gene pair** | Ka | Ks | Ka/Ks |
| --- | --- | --- | --- |
| PvLAC2/PvLAC8 | 0.3057265 | 2.7352818 | 0.1117715 |
| PvLAC2/PvLAC20 | 0.0937525 | 0.7940509 | 0.1180686 |
| PvLAC7/PvLAC23 | 0.0825998 | 0.5723188 | 0.1443248 |
| PvLAC8/PvLAC22 | 0.1056918 | 0.6304005 | 0.1676582 |
| PvLAC8/PvLAC20 | 0.2943437 | 1.7267344 | 0.1704626 |
| PvLAC15/PvLAC21 | 0.235308 | 1.8415973 | 0.1277739 |
| PvLAC15/PvLAC23 | 0.1905893 | 1.4884644 | 0.1280443 |
| PvLAC16/PvLAC25 | 0.2845188 | 2.0161035 | 0.1411231 |
| PvLAC18/PvLAC28 | 0.2259491 | 1.5699494 | 0.1439213 |
| PvLAC21/PvLAC23 | 0.2329614 | 2.3669209 | 0.0984238 |
| PvLAC28/PvLAC29 | 0.1224681 | 0.7925049 | 0.1545329 |

**Attached map**


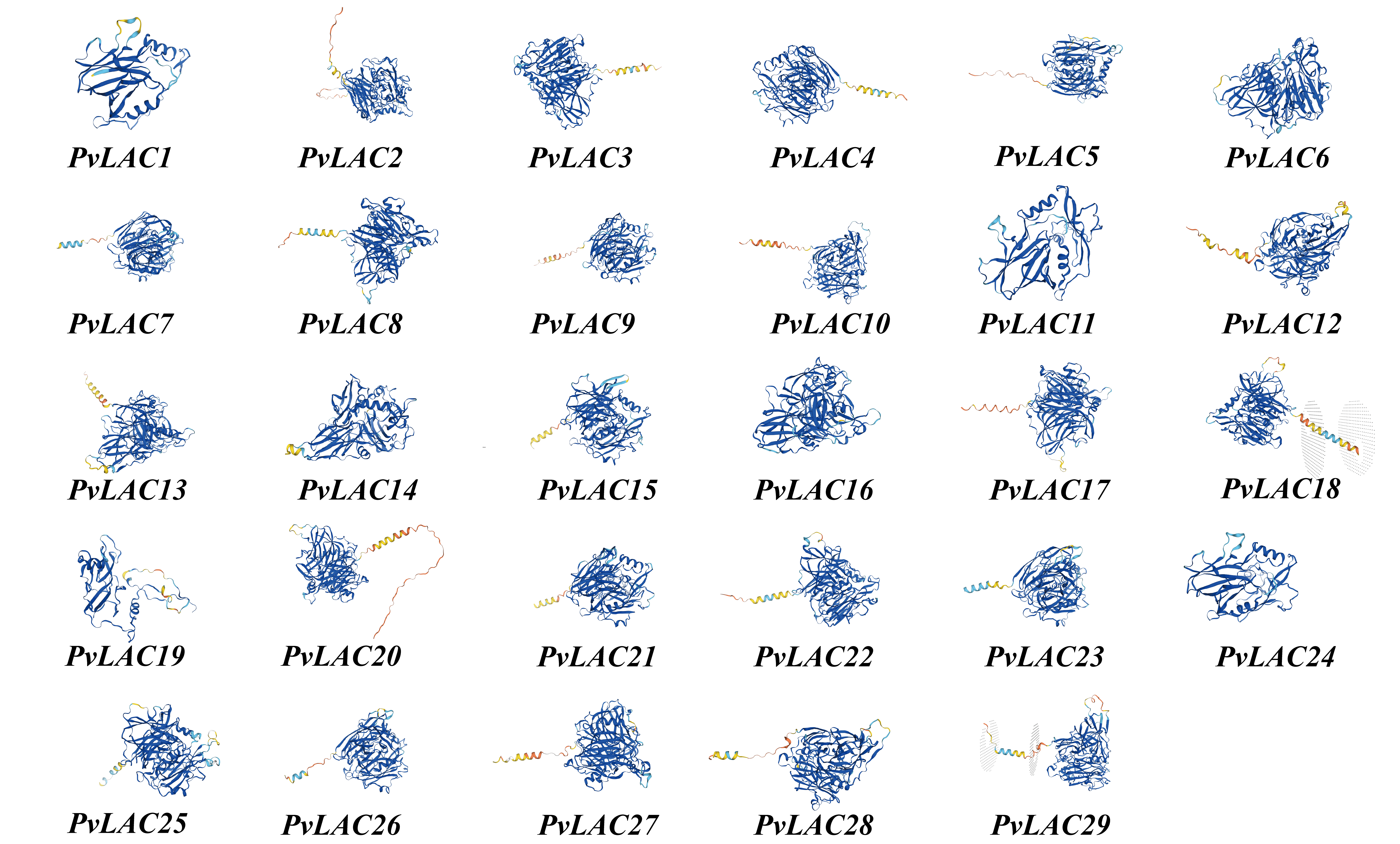


**Fig.S1** 3D structure prediction of PvLAC protein





**Fig.S2** Species evolution tree of laccase gene family. A: Expansion and contraction of LAC gene family members from a species perspective B: acquisition and loss of LAC gene family members from a genetic perspective


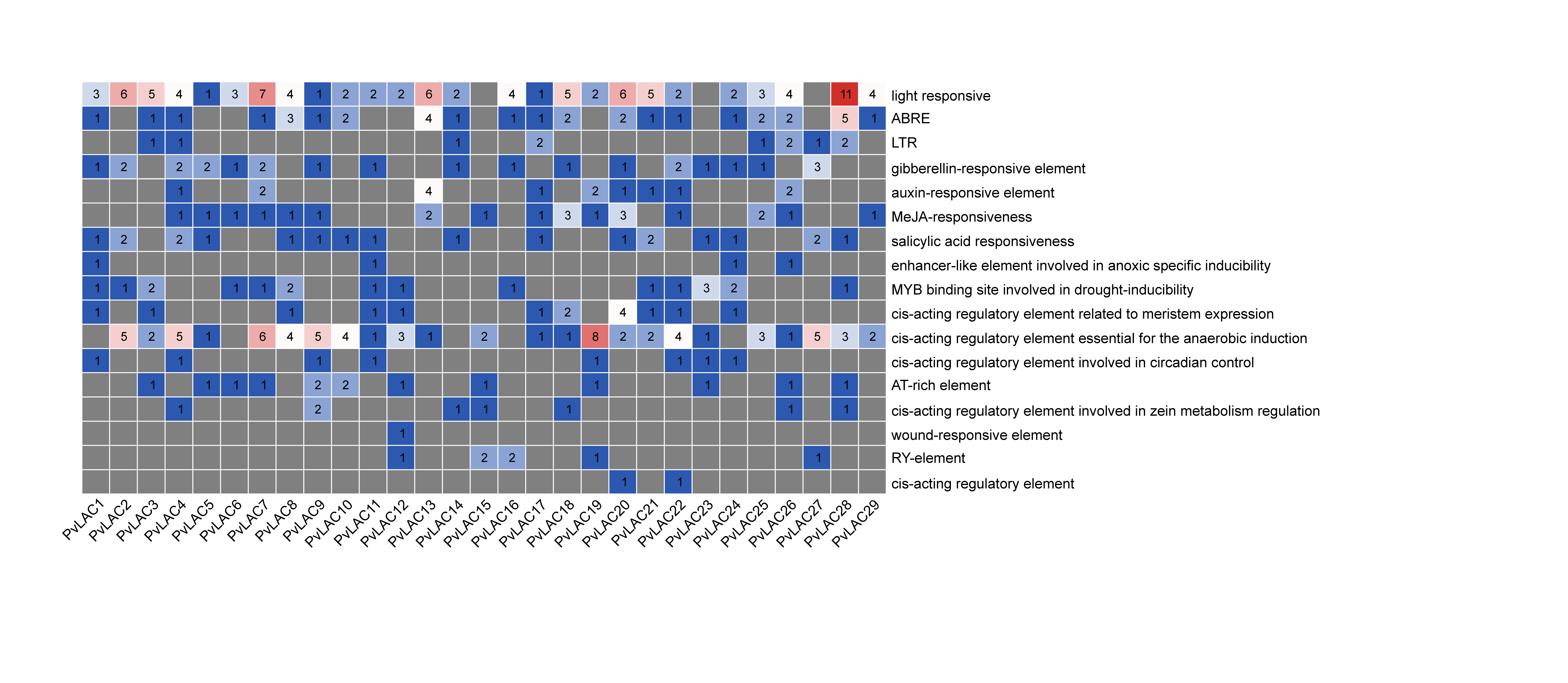


**Fig.S3** PlantCARE. *Cis*-acting elements in the promoter region of *PvLACs*-s. (The number represents the number of existence)


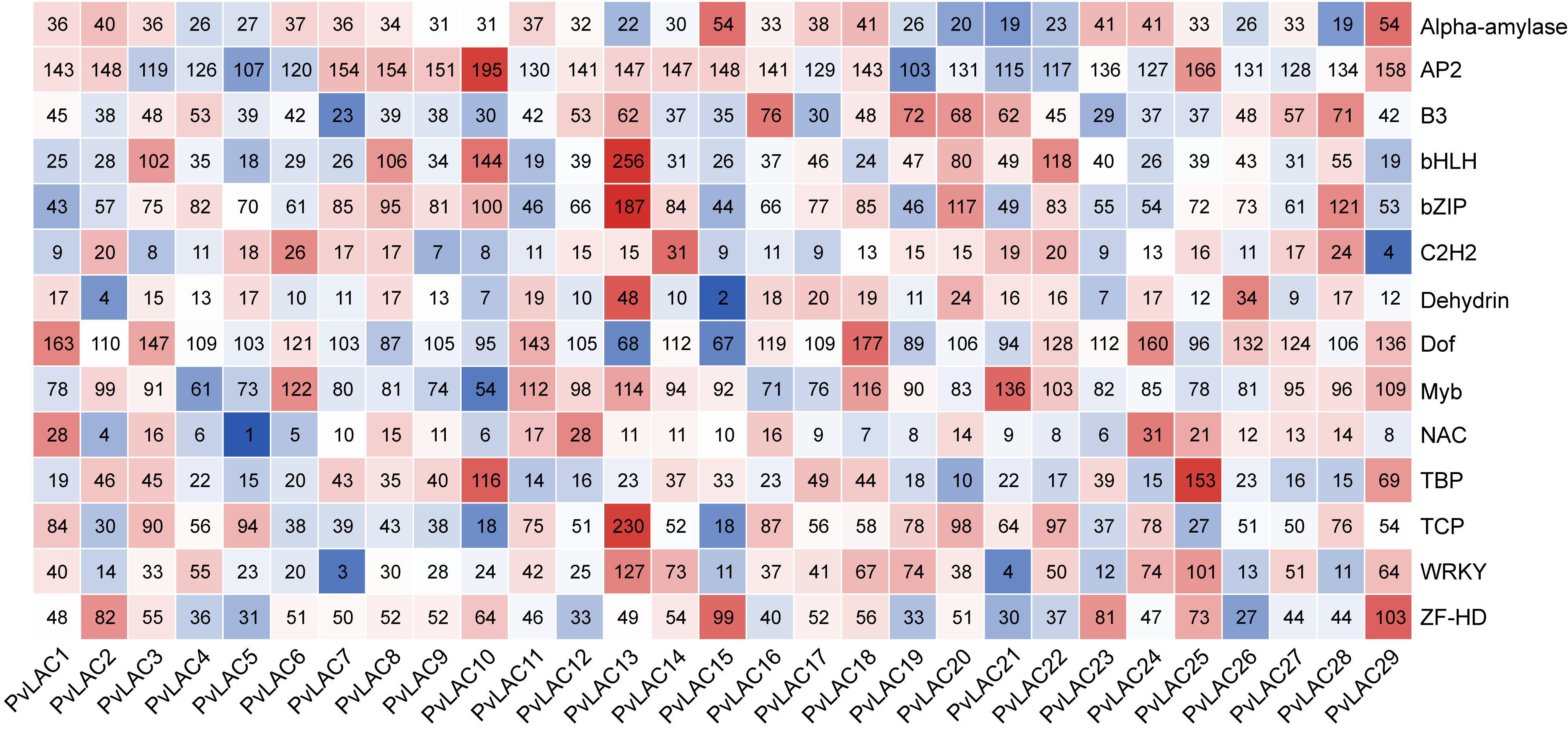


Fig.S4 PlantPAN4.0. *Cis*-acting elements in the promoter region of *PvLACs*-s. (The number represents the number of existence)


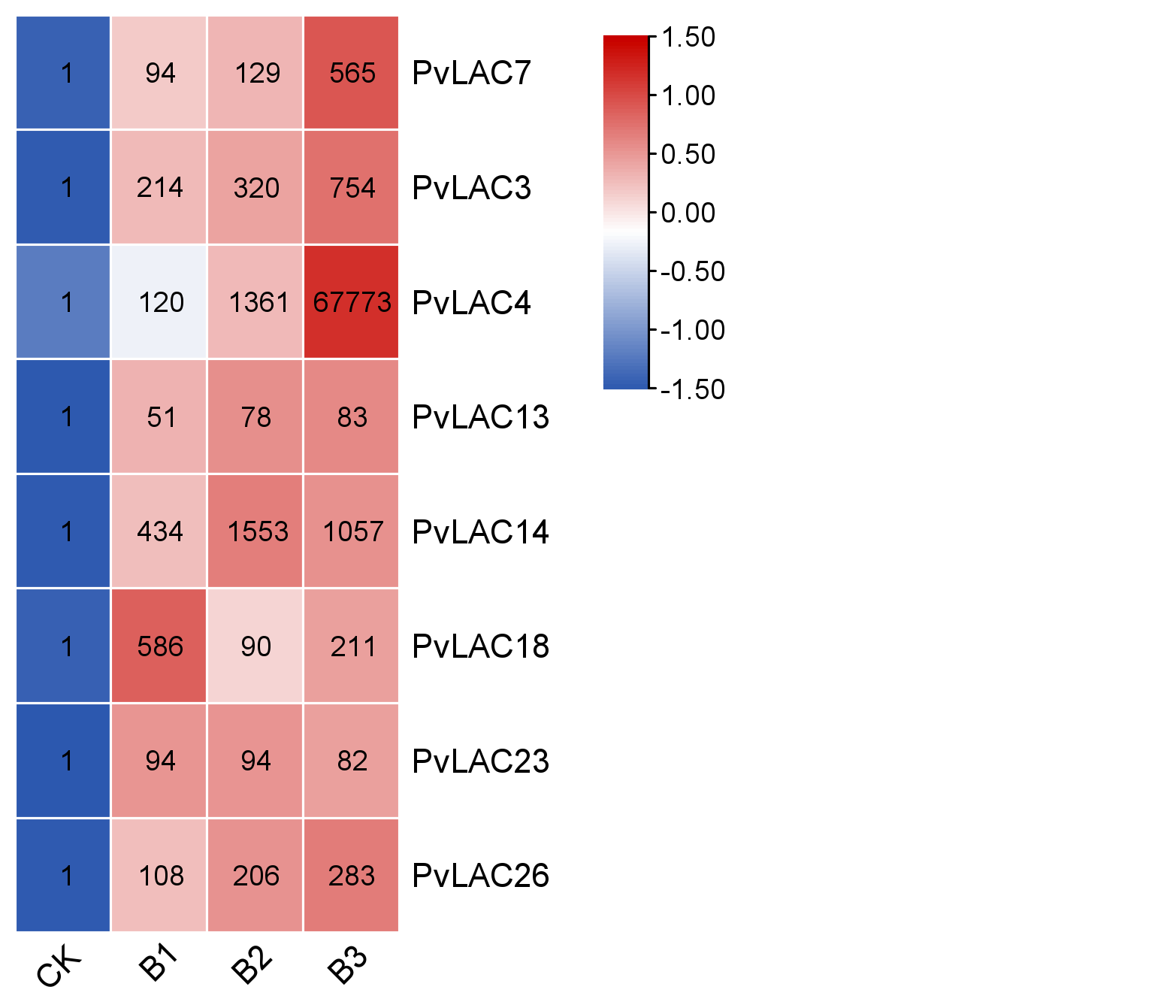


**Fig. S5** Expression profiles of 8 PvLACs under treatment with three concentrations of glyphosate, B1:100ug/Kg,B2:150ug/Kg,B3:200ug/Kg.

**Fig. S6** Phenotypes of PvLAC3, PvLAC4, PvLAC7, PvLAC14 silenced plants under cold stress.

**Other supplementary materials**

qRT-PCR primer

| PvLAC3 | **Forward primer** | GCACAATGACGTGGAAGCTG |
| --- | --- | --- |
|  | **Reverse primer** | AGGGCAGCGTTGATGATTCT |
| PvLAC4 | **Forward primer** | ACGATCAATGGGAAGCCAGG |
|  | **Reverse primer** | TCGTCATTGAGGGCAGCATT |
| PvLAC7 | **Forward primer** | GGAAGCTATGGTTCGCCACT |
|  | **Reverse primer** | GCATAGATGGTGGGACCTGG |
| PvLAC13 | **Forward primer** | CTTCTGGTGGATGGGTTGCT |
|  | **Reverse primer** | TAAGTCCCCAGGTTGTGTGC |
| PvLAC14 | **Forward primer** | CTGGCCTTGGCACTTTTGAC |
|  | **Reverse primer** | GTCGTTGAGTGCAGGGAGAA |
| PvLAC18 | **Forward primer** | AAGGTGAAGCCTGGGAAACC |
|  | **Reverse primer** | AGCTTCCACAACAGTGAGGG |
| PvLAC23 | **Forward primer** | TCCACCATCACAACCCTCAC |
|  | **Reverse primer** | ACAAGGGTTGACTCCAAGGC |
| PvLAC26 | **Forward primer** | AGTTCCCAGGACCCACTCTT |
|  | **Reverse primer** | TCCATCAGCCCAACCAGTTC |

Primers for constructing VIGS vector

| PvLAC3 | **Forward primer** | TCTAGTCAAGGCCAACCAAGT |
| --- | --- | --- |
|  | **Reverse primer** | CATGTAGGGCAAGAATTCTGGG |
| PvLAC4 | **Forward primer** | GTGGGGCAAGAATTCTGGC |
|  | **Reverse primer** | CGTTCTGGTCCAAGCTAACCAA |
| PvLAC7 | **Forward primer** | TGAGACAAGTGAGAACAGGTTGGG |
|  | **Reverse primer** | GTGTTTCCTGGTTGAACATCCAAC |
| PvLAC14 | **Forward primer** | ACAGCTTCGATCAGGGTGG |
|  | **Reverse primer** | TTTGGCCCTCCACCAGTTT |

Sequence selected for VIGS

>PvLAC3

TCTAGTCAAGGCCAACCAAGTAGCAGGAAGATACTTCATGGCTACAAGGACCTTTATGGATGCACCGATCCCAGTTGACAACAAATCCGCCACAGCAATATTCCAATACAAAGGCATTCCAAACACTGTCCTCCCTTTTCCTCCTTCTCTTCCTGCTGCCAATGACACACCTTTTGCTTTGAGTTACAACAACAAAATAAGAAGCCTAAACTCTCCTCAGTACCCCGCTAATGTTCCACTCGAAGTTGATCGAAACCTCTTTTACACCATCGGTTTGGCCCAGAATTCTTGCCCTACATG

>PvLAC4

CGTTCTGGTCCAAGCTAACCAAGTTGCAGGTAGATACTTCATAGCCACCAGGGCCTTTATGGATGCTCCAATTCCAGTTGACAACAAAACTGCCACAGCTATTCTCCAATACAAAGGCATCCCAAACACTGTCCTCCCCATCCTTCCACAGTTGCCGGCTAGCAATGACACACGTTTTGCTTTGACTTACAACAAGAAACTAAGGAGCCTGAACTCTGCTCGTTACCCTGCTAATGTTCCTCTCAAAGTTGATAGAAACCTTTTCTACACTATTGGTTTAGGCCAGAATTCTTGCCCCAC

>PvLAC7

TGAGACAAGTGAGAACAGGTTGGGCTGATGGGCCAGCATACATAACACAATGCCCAATTCAACCGGGTCAGGCCTATGTCTACAACTTTACCCTTACAGGCCAGAGAGGCACACTTTGGTGGCATGCACATATCCTCTGGCTTAGGGCCACTCTCCATGGAGCCTTGGTCATCCTACCCAAGCTTGGAGTACCTTACCCTTTTCCCAAACCAAATATGGAACAAGTTATCATACTAAGTGAATGGTGGAAATCAGACACTGAGGCTGTAATAAATGAAGCTTTGAAATCTGGTTTGGCACCTAATGTCTCTGATGCTCACACAATCAATGGTCATCCAGGACCTGTCCAAGGCTGTGCTTCACAAGAAGGATTTAAGTTGGATGTTCAACCAGGAAACAC

>PvLAC14

ACAGCTTCGATCAGGGTGGGCTGATGGGCCAGCATATGTGACTCAATGCCCCATCCAAACTGGTCAAACTTATGTTTACAATTACACCATTACTGGCCAAAGGGGAACACTGTTTTGGCATGCCCATATATCATGGCTAAGAGCAAGTCTTTATGGTCCTCTCATCATTCTTCCCAAGCTCAATGCTCAATATCCTTTTGCTAAACCCCACAAGGAAGTTCCTATGGTGTTTGGAGAATGGTGGAATGTAGATCCTGAGGCAGTCGTAACCCAGGCCACGCAAACTGGTGGAGGGCCAAA

Plasmid map of PNC-TRV2 (pTRV2 )


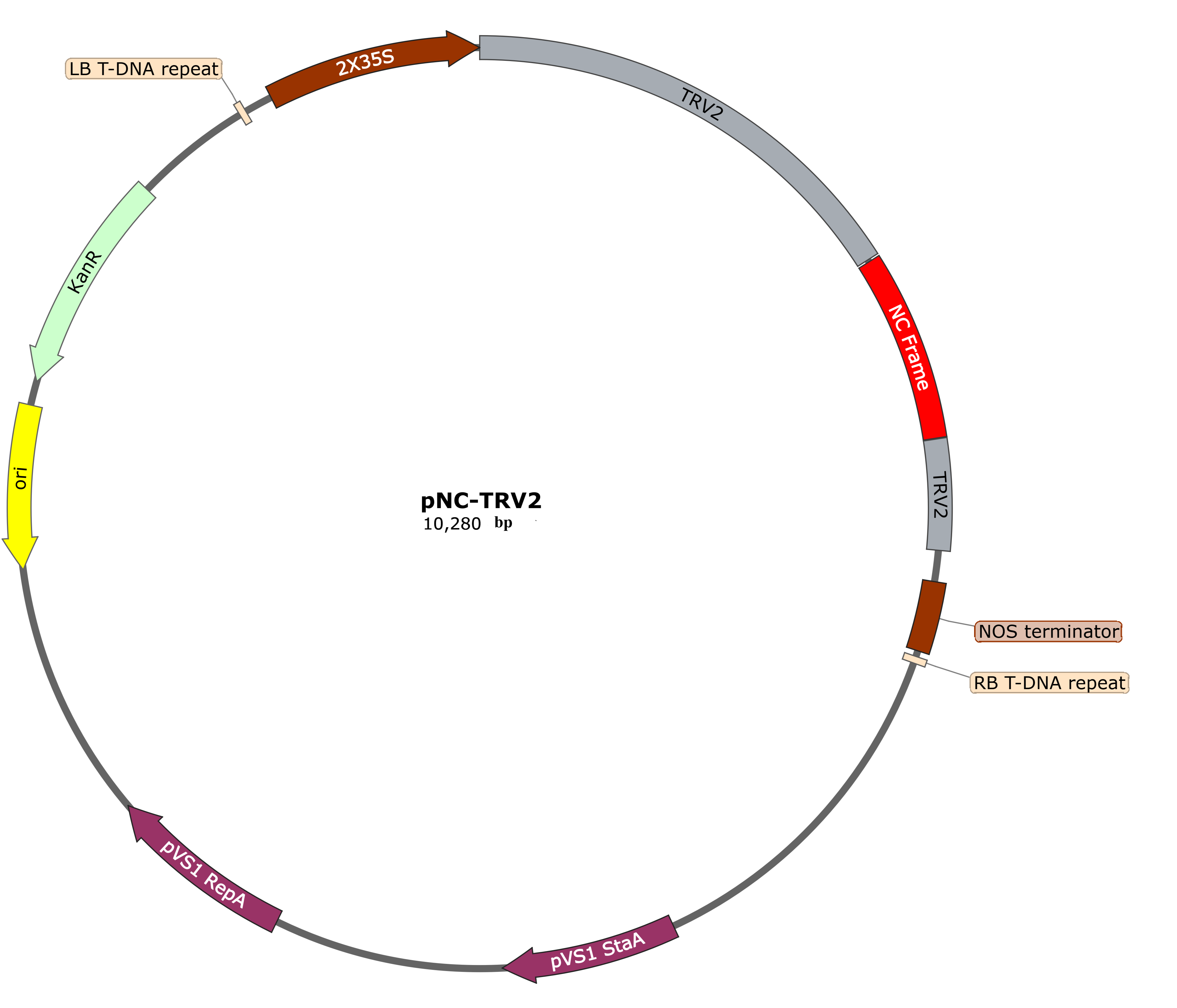


Gel electrophoresis image





PCR amplification of recombinant pTRV2 plasmid. The TRV1 and TRV2 vectors were obtained from NC Biotech. The pTRV2-*PvLAC3*, pTRV2-*PvLAC4*, pTRV2-*PvLAC7*, and pTRV2-*PvLAC14* vectors were constructed using the Nimble Cloning kit and the pNC-AEnTopo blunt-end cloning kit from NC Biotech. PCR analysis was performed to confirm the presence of pTRV2-*PvLAC3*, pTRV2-*PvLAC4*, pTRV2-*PvLAC7*, and pTRV2-*PvLAC14*. The first well of each group uses recombinant plasmids as templates, the second well uses cDNA from ordinary beans as a positive control template, and the third well uses pTRV2 plasmid as a negative control template.

**A**


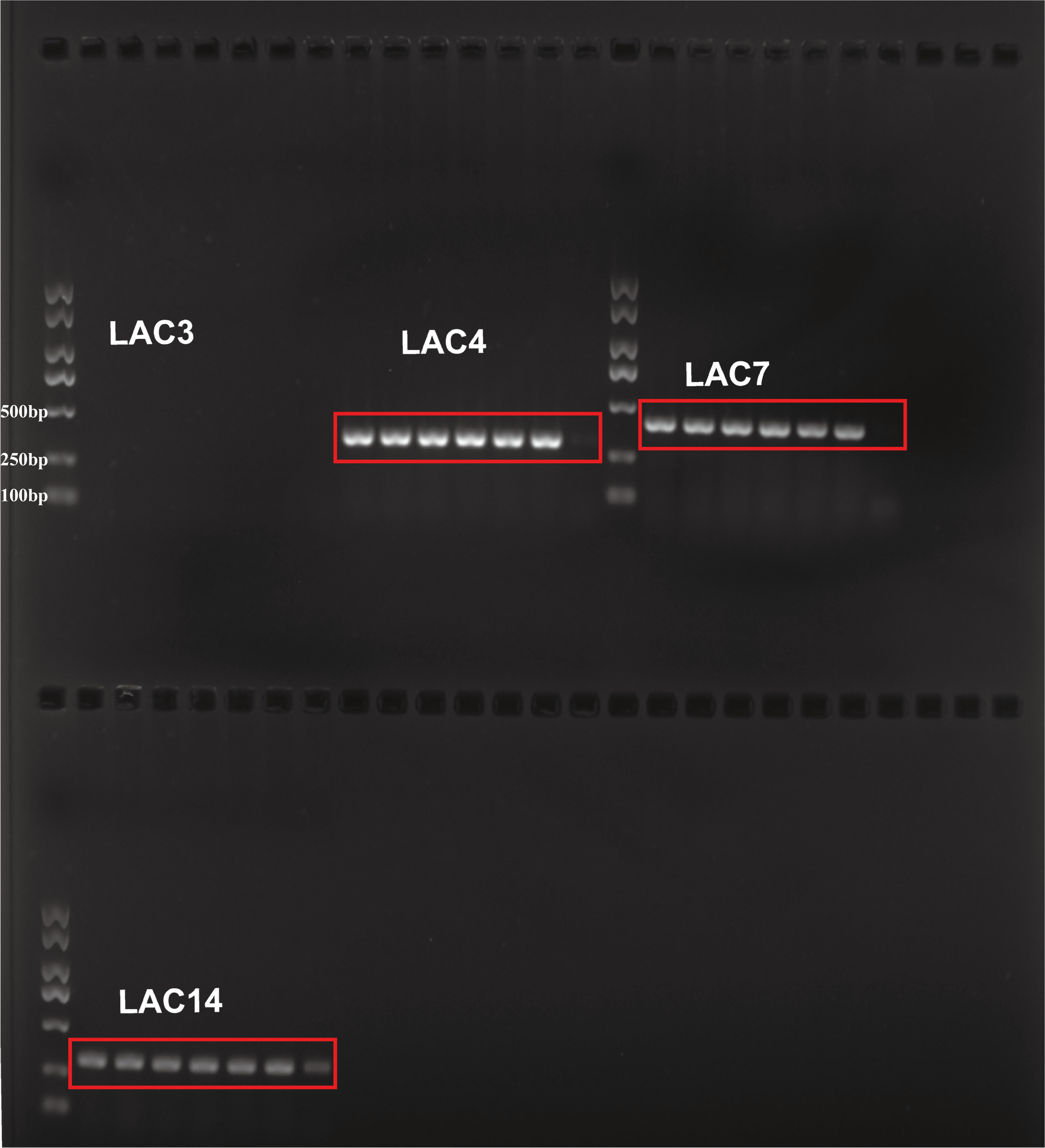


**B**


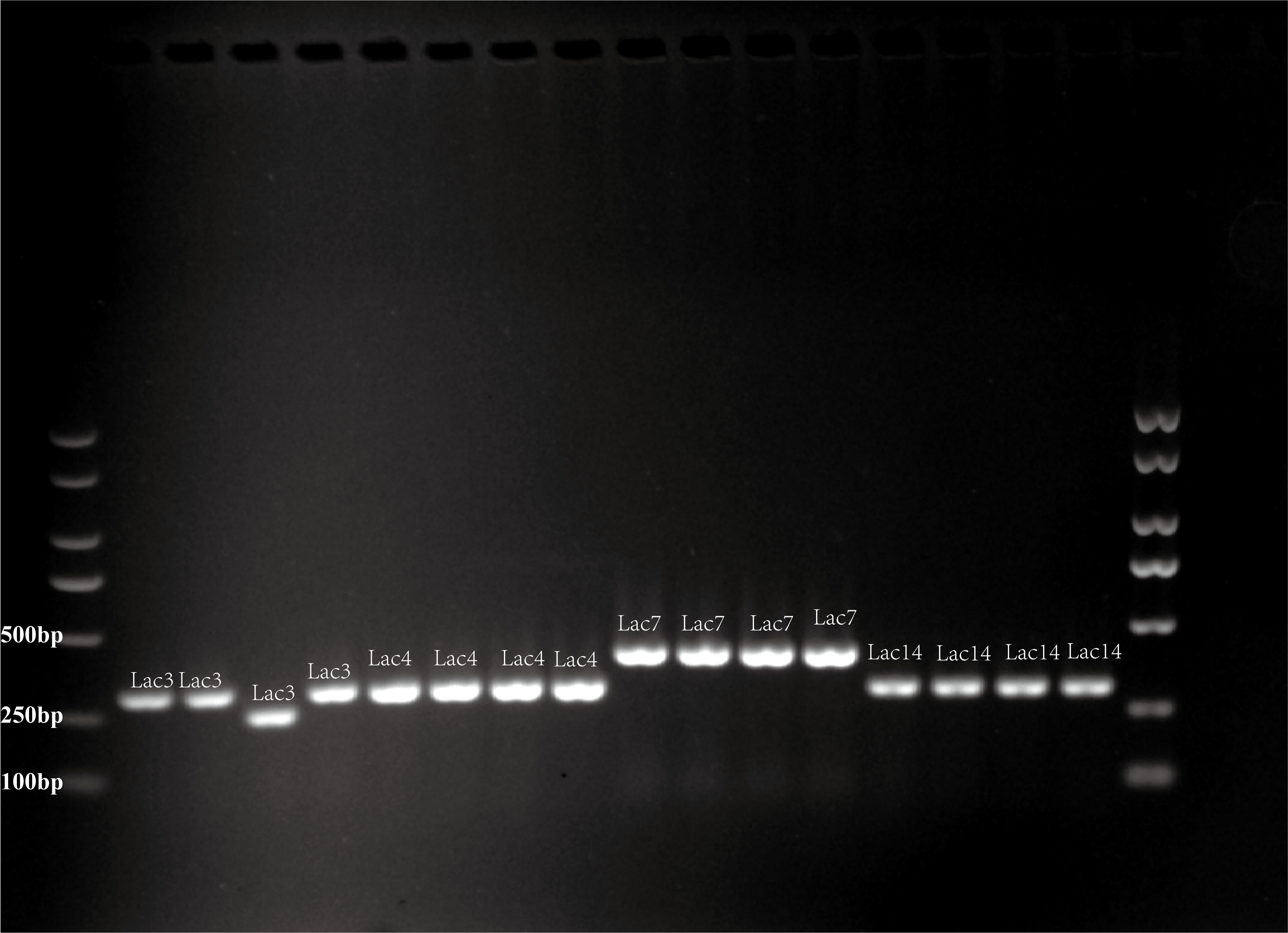


Agrobacterium culture PCR identification. A: The pTRV1, pTRV2, pTRV2-*PvLAC3*, pTRV2-*PvLAC4*, pTRV2-*PvLAC7*, and pTRV2-*PvLAC14* vectors were transformed into the Agrobacterium tumefaciens strain GV3101 using heat shock. Agrobacterium culture PCR identification was performed, with the last well of each group serving as a negative control (using pTRV2 Agrobacterium as template). B: During the initial introduction of *PvLAC3*, it was introduced into the wrong vector. The second attempt at introduction was successful.
